# Individual Preference Framework or Group Preference Framework? Which Will Regulate the Impact Path of Product Facilities on Residents’ Waste-Sorting Behavior Better

**DOI:** 10.3390/ijerph17072324

**Published:** 2020-03-30

**Authors:** Feiyu Chen, Fang Wang, Jing Hou

**Affiliations:** 1 School of Management, China University of Mining and Technology, No.1 Daxue Road, Xuzhou 221116, China; chenfeiyu@cumt.edu.cn (F.C.); 09163486@cumt.edu.cn (F.W.); 2 Business School, Jiangsu Normal University, No.101 Shanghai Road, Xuzhou 221116, China

**Keywords:** waste-sorting behavior, individual preference framework, group preference framework, product facilities

## Abstract

To effectively deal with the waste management problems faced by cities, it is of great significance to promote the sorting and recycling of municipal solid waste. Given the correlation between individual behavior and psychological preferences and external situations, this study explored the mechanism of individual preference framework and group preference framework in the impact path of product facilities on residents’ waste-sorting behavior. Based on a questionnaire survey (*N* = 1505), combined with correlation analysis, difference analysis, hierarchical regression analysis, sensitivity analysis, and other methods, the study found that differences in residents’ age, education background, and monthly income lead to differences in residents’ sorting behaviors, and individuals of young age and low monthly income have higher sorting behaviors than others. Interestingly, highly educated individuals did not show high sorting behavior. Both individual preference and group preference frameworks play a regulating role in the influence path of product facilities on waste-sorting behavior, but a group preference framework (including family preference, organizational preference and social preference) plays the more significant regulating role. Additionally, social preference variables are the most prominent regulatory factors and have a greater “amplifier” effect in the impact of product facilities on waste-sorting behavior. Based on these findings, this study identifies the corresponding policy implications.

## 1. Introduction

In recent years, the problem of municipal solid waste (MSW) globally has become increasingly serious. In 2017, the domestic waste output of 202 large and medium-sized cities in China was 201,944,000 tons, which has aroused widespread concern from the government and all sectors of society [1]. MSW mainly comes from homes, public places, commercial departments, and public institutions [2]. From the perspective of component, MSW includes paper, plastic, glass, batteries, metal, cloth, lime soil, and fallen leaves. The proportion of each component varies in different cities, seasons, and places [2]. However, the rate of MSW generation is much faster than the rate at which it is recovered, reused, or assimilated safely back into the environment. Since 2000, China has started pilot projects to promote waste sorting and recycling in some cities. Many types of waste sorting facilities were designed and used to guide residents sorting properly in the area of public, work, and household, such as colored sorting trash, identification sorting bin, and integral dustbin [2,3]. But the projects made little success, MSW is hindering the process of sustainable development in Chinese cities [4]. Therefore, the key to solve the problem of waste management is to guide urban residents to participate in waste sorting activities and improve the effectiveness of MSW sorting and recycling.

Combing the existing relevant studies, some scholars have shown that the lack of effective sorting and recycling facilities is one of the main obstacles preventing Chinese residents from sorting and reusing most recyclable wastes [4]. The availability, accessibility, and facility convenience of sorting facilities all affect residents’ waste-sorting behavior [5,6,7,8]. In addition, product characteristics are also important factors influencing individual sorting behavior [9]. Product facilities which indicate product characteristics and waste management facilities [10], have a promoting effect on residents’ waste-sorting behavior. However, both Theory of Planned Behavior and Situated Cognition points out that individual behavioral decisions are not only influenced by situational factors, but also by the interaction of their own psychological characteristics [11,12]. Individual waste-sorting behavior may also be so influenced by this interaction.

Psychological preference is an important aspect of individual psychological characteristics, and refers to a psychological tendency to choose things that meet one’s “taste” [13,14]. On the one hand, psychological preference is reflected in one’s daily pursuit of time, efficiency, quality of life, etc. [15]. On the other hand, it is also reflected in one’s normative preference for things, people, etc., [16,17]. For the former, scholars have shown that individuals’ pursuit of life comfort and preference for travel time will affect their sorting behavior [18,19,20]. For the latter, personal normative preferences (specifically based on personal sense of moral obligation, values, etc.) and subjective normative preferences (referring to the social pressure felt by individuals from other people or groups) under the framework of the Norm Activation Model are considered important factors driving individuals to conduct waste sorting and recycling [21,22,23,24,25,26].

Based on these findings, combined with the goal framework theory [27] and from the perspective of individuals, this study divides psychological preferences into individual preference framework variables and group preference framework variables, builds up a four-dimensional structure for product facilities, individual preference framework, group preference framework, and waste-sorting behavior, to explore and compare the mechanism of the two sets of variables in the influence path of product facilities on waste-sorting behavior. Individual preference framework refers to the preference expressed by the individual due to the internal itself; group preference framework refers to the preference expressed by the individual due to the pressure of group environment.

Given the correlation between individual behavior and psychological preferences and external situations, this study aims to explore the mechanism of individual preference framework and group preference framework in the impact path of product facilities on residents’ waste-sorting behavior. By selecting six first-tier cities in the north, East, and south of China, a questionnaire survey is conducted. Combined with correlation analysis, difference analysis, hierarchical regression analysis, sensitivity analysis, and other methods, the relationship mechanism among product facilities, preference framework, and waste-sorting behavior is explored and verified.

## 2. Literature Review 

### 2.1. Related Research on the Influence of Product Facilities on Waste-Sorting Behavior

Many scholars believe that the environmental facilities and environmental products are important conditions that affect the environmental behavior. For example, Santos [28] showed that the public’s environmental behavior is affected by the environmental situation; although people hold positive environmental values, if green products are not widely available, residents will still exhibit non-environmentally friendly purchasing behavior. Chan’s research on green purchasing in mainland China also supports this view [9]. In addition, Barr [29] found in comparing residents’ waste sorting and recycling behavior in the United States and the United Kingdom that external conditions (e.g., whether to set up recycling bins and other community public facilities and whether to have convenient locations) have a significant impact on residents’ waste sorting and recycling behavior. Some scholars have pointed out that necessary infrastructure [30], convenient sorting facilities and collection services [6], and increased accessibility to waste collection facilities [31] will promote the public’s active participation in waste sorting activities. These studies have verified from different perspectives that the combined effect of product characteristics and waste management facilities has a significant impact on the expression of environmental behavior. Waste-sorting behavior is a typical environmental behavior, and in the present study it was hypothesized that this combination will also have a significant impact on the residents’ waste-sorting behavior.

### 2.2. Related Research on the Influence of Individual Preference Framework and Group Preference Framework on Waste-Sorting Behavior

In many cases, residents are also influenced by their preferences when they participate in environmental activities. In the study of low-carbon traffic behavior, Bowman and Ben-Akiva found that travelers’ time preference significantly affects their choice of travel modes [18]. Anker-Nilssen [19] analyzed the energy use statistics of Norwegian households from 1973 to 1990, and found that residents’ increased demand for living comfort is the most important reason for the increase of household energy use. Gatersleben et al. [32] claimed that wasting behavior is more likely to occur in the group who like living in big houses. Some scholars believe that group preference will also have an impact on residents’ environmental behavior. For example, Zhang et al. [33] showed that when an individual’s friends actively participate in waste sorting, the individual is more willing to make efforts to participate in waste sorting. Shaw [34] claimed residents’ willingness to recycle is affected by neighbors, especially the nearest neighbors. Valle et al. [35], Miliute-Plepiene et al. [36], and Siegmar et al. [37] found that a person’s sorting and recycling behavior is also influenced by social norms held by other people and social groups that he or she thinks are important to him or her. Antonella et al. [38], Terrier and Marfaing [39], and Mintz et al. [40] have also verified the predictive effect of social culture and environmental stress on public pro-environmental behavior. 

According to the relevant literature, this study defined two key concepts from the perspective of individuals. First, the individual preference for scale and scene, time urgency and efficiency, quality of life and comfort in the process of daily life was defined as the individual preference framework (including preferences for quantity: residents’ pursuit of scale and scene in the process of daily consumption; preferences for rhythm: residents’ pursuit of time urgency and efficiency in daily life and work; preferences for quality: residents’ pursuit of quality of life in the process of daily life). Second, the tendency that individuals show because of the pressure brought by energy conservation and environmental ethos and moral standards in the family, organization, society, and the external social public was defined as the group preference framework (including family preference: the tendency of individuals to be environmentally friendly because they feel the environmental protection atmosphere in their families and the pressure brought by their relatives; organizational preference: the tendency of individuals to be environmentally friendly because they feel the environmental atmosphere in the organization and the pressure from colleagues; social preference: the tendency of individuals to be environmentally friendly due to the environmental protection atmosphere in society and the pressure brought by the public). These two concepts were examined to determine whether both have a significant effect on residents’ waste-sorting behavior. 

### 2.3. Relationship Between Preference Framework and Waste-Sorting Behavior

In the study of environmental behavior, scholars often use individual internal factors and environmental situational factors as independent variables of environmental behavior and analyze the direct causal relationship between them. These studies have included that residents conduct of waste-sorting behavior is influenced not only by moral obligations, values, and economic interests [41,42], but also by family members [26], organizational norms [37], and public attitudes [27]. However, some scholars believe that these influences are not only simple one-way interactions, but rather that some factors also have a regulating effect. For example, Guagnano et al. [43] pointed out in the attitude-situation-behavior theory that environmental behavior is the result of the interaction between environmental attitude variables and situational factors. Hong et al. [44] claimed that government incentive measures and psychological factors play an important role in promoting energy-saving behaviors, and have a significant positive regulating effect on attitude and energy-saving behaviors. Zhang et al. [45] found that good publicity of environmental policies may further promote the relationship between consumers’ psychological needs and environmental awareness. Babazadeh et al. [46] summarized the interactive effects of external context variables and internal characteristic variables on individual waste-sorting behavior through qualitative research.

In addition, many scholars have verified the regulating effect of preference on other behavioral variables. For example, Zhang et al. [47] found that when a person has a stronger sorting preference, the positive correlation between work-family conflict rated by the person’s spouse and the person’s work-family guilt is stronger. Mohammad [20] showed that the concentration of residents based on neighborhood preferences moderates the association between certain characteristics of density measurements and tourist behavior. The present study further investigated the regulating effect of preference framework variables on the behavioral mechanism during waste-sorting activities.

## 3. Research Design and Implementation

### 3.1. Research Method

Based on the literature review, there is a certain relationship between residents’ waste-sorting behavior and product facilities, preference framework. This study uses correlation analysis, difference analysis, hierarchical regression analysis, and sensitivity analysis to explore the role of preference framework in the path of product facilities’ impact on residents’ waste-sorting behavior. Specifically, correlation analysis is used to verify whether product facilities have an impact on residents’ waste-sorting behavior; difference analysis is used to compare the impact of individual differences on residents’ waste-sorting behavior; hierarchical regression analysis is used to explore the mechanism of preference framework in the path; sensitivity analysis is used to compare the difference between individual preference framework and group preference framework in the path.

### 3.2. Scale Design and Pre-Survey

Based on existing product facilities, individual preference frameworks, group preference frameworks, and relevant mature scales of urban residents’ waste-sorting behavior, this study revised and developed relevant measurement items by combining descriptors of the actual situation, supplemented by expert evaluation and other methods, to obtain an initial scale to measure individual and group preference networks and product facilities. To test the feasibility of the scale, a preliminary study was conducted in October 2018, during which questionnaires were collected from 256 randomly selected people in different regions of China through an online survey (of which 203 were completed fully and correctly). The reliability and validity of the questionnaire were analyzed, and the questions were revised to form a final questionnaire for subsequent use in the full study.

The final questionnaire consisted of five parts (see Appendix A). The first part collected demographic information from respondents, which was used to test the distribution of samples and help analyze responses. This information mainly included the respondent’s gender, age, education background, political status, family monthly income level, and household chores. The second part contained the scale of product facilities, which was used to measure the facility specification of the environment in which each respondent lived. The third part contained the scale of the individual preference framework, which was used to measure the individual preferences of respondents. The fourth part contained the scale of the group preference framework, which was used to measure the overall preference of the respondents in their environment. The fifth part consisted of the scale of waste-sorting behavior, which was used to measure a respondent’s intention and implementation of waste-sorting behavior in real life. Participants were asked to use Likert five-point scales to evaluate the agreement of the descriptions, where “not conformed” = 1, “not quite conformed” = 2, “not sure” = 3, “somewhat conformed” = 4, “quite conformed“ = 5. Sample items of the four scales are shown in Table 1.

### 3.3. Formal Investigation and Sample Analysis

The formal investigation was conducted in November 2018. The questionnaire was distributed on a large scale throughout China’s first-tier cities such as Beijing, Shanghai, Guangzhou, Shenzhen, Nanjing, and Hangzhou. Data were collected through a combination of online questionnaires and paper questionnaires. To ensure sample population was representative of the general population, the sample structure was pre-stratified based on the actual population characteristics, such as gender, age, education background, and monthly income. Subsequently, questionnaires were randomly distributed to urban residents through online platforms (e-mail, WeChat, QQ and other social media). The paper questionnaires were distributed in person by research team members visiting major shopping malls, libraries, museums, fast food restaurants, and residential areas in cities.

A total of 920 paper questionnaires were distributed and 738 valid questionnaires (82.00%) were recovered. A total of 986 online questionnaires were issued, and 767 valid questionnaires (77.79%) were recovered. A combined total of 1906 questionnaires were issued and 1505 valid questionnaires were recovered, yielding an effective recovery rate of 78.96%. The sample distribution is shown in Table 2. It can be seen that the sample has a certain sample size (*n* ≥ 59) under different ages, genders, education backgrounds, and monthly incomes, and the ratio distribution is almost consistent with the demographic variables distribution in Chinese cities (refer to China statistical yearbook 2018). The sample distribution was deemed to be reasonable and representative. SPSS Statistics for Windows 17.0 (SPSS, Inc., Chicago, IL, USA) and AMOS 17.0 (IBM, Armonk, NY, USA) were used to analyze the questionnaire data.

### 3.4. Reliability and Validity Tests

Cronbach’s coefficient was used to measure the reliability of the questionnaire. The coefficient was determined using SPSS 17.0 software. The results show that, the Cronbach’s coefficient of the product facilities, the individual preference framework and its dimensions, the group preference framework and its dimensions, and waste-sorting behavior all exceeded 0.7, and most exceeded 0.8. These values were within an acceptable range, confirming that the scales were reliable.

The validity test mainly examined the content validity and structural validity of the questionnaire. Based on existing widely used scales, the questionnaire developed for this study was obtained through expert consultation and repeated discussion and modification by members of the research team; therefore, it was presumed to have high content validity. “Bartlett’s test” showed that the Kaiser-Meyer-Olkin (KMO) values of each scale exceeded 0.65, and most exceeded 0.8 (KMO _individuals_ = 0.848, KMO _group_ = 0.898, KMO _product facilities_ = 0.690, and KMO _waste sorting behavior_ = 0.898). The significance level of Bartlett’s test was 0.000, indicating that each scale passed the preliminary validity test and was suitable for factor analysis.

Next, AMOS17.0 software was used to conduct confirmatory factor analysis on individual preference framework and group preference framework variables using maximum likelihood estimation. Cross-loading was not allowed, and the fixed variance was used to set the model. The data showed that the goodness-of-fit test parameters of the individual preference three-factor model all reached the acceptable range (χ^2^ = 2935.582, CDMIN/DF = 3.255, RMSEA = 0.032, GFI = 0.916, NFI = 0.906, CFI = 0.941, TLI = 0.932). The goodness-of-fit indexes of the group preference three-factor model were even better than that of the individual preference model, and the model had better structural validity (χ^2^ = 2633.987, CDMIN/DF = 4.362, RMSEA = 0.044, GFI = 0.925, NFI = 0.905, CFI = 0.899, TLI = 0.887). It assumes that the validity and reliability tests have absorbed all the assumptions such as heteroskedasticy, homoskedasticy, stationarity, and auto-correlation that could have impacted the regression.

## 4. Results

### 4.1. Correlation Analysis

**Figure 1:**
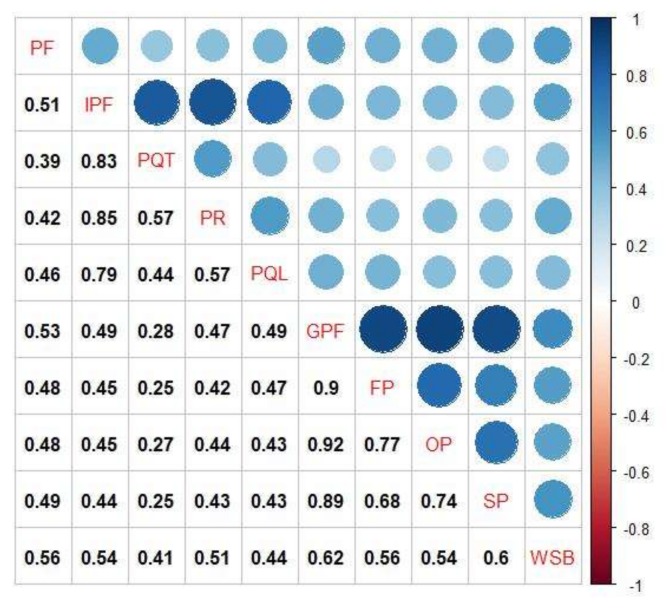
Correlation coefficient matrix of each variable (N = 1505). Note: PF indicates product facilities, IPF indicates individual preference framework, PQT indicates preferences for quantity, PR indicates preferences for rhythm, PQL indicates preferences for quality, GPF indicates group preference framework, FP indicates family preference, OP indicates organizational preference, SP indicates social preference, WSB indicates waste-sorting behavior.

Correlation analysis showed that waste-sorting behavior and product facilities had a significant positive correlation (*r* = 0.559, *p* < 0.01) (Figure 1). Waste-sorting behavior was significantly positively correlated with the individual preference framework and its dimensions, as well as with the group preference network and its dimensions. Among them, the correlation between waste-sorting behavior and the group preference framework and its dimensions (*r* = 0.610, *p* < 0.01) was higher than that of the correlation with the individual preference framework. Among the dimensions of the group preference network, social preference was most highly correlated with waste-sorting behavior (*r* = 0.596, *p* < 0.01).

### 4.2. Analysis of Differences in Residents’ Waste-Sorting Behavior in Terms of Gender, Age, Education Background and Monthly Income

SPSS 17.0 was used to explore the specific differences of residents’ waste-sorting behavior in terms of gender, age, education background, and monthly income, based on the independent sample t-test, one-way variance test, and comparison of mean values. The independent sample t-test was conducted on gender, the one-way variance test was conducted on age, educational background, and monthly income variables, and the average score of waste-sorting behavior of each respondent group was calculated. The results of these data analyses are shown in Table 3.

In the results of the independent sample t-test, the *p*-value of the gender variable exceeded 0.05, indicating that there was no significant difference in the waste-sorting behavior of urban residents in terms of gender. In the one-way variance test, the *p*-value of the age variable was 0.045, the *p*-value of the education background variable and the monthly income variable were 0.000; because all of these values were less than 0.05, the waste-sorting behavior of urban residents was significantly different among respondents of different ages, and among those having different education backgrounds and monthly incomes.

Specifically, in terms of age, residents under 17 exhibited the highest mean scores for waste-sorting behavior, which were much higher than those for residents of other ages. Urban residents aged 18–25 had the lowest mean scores for waste-sorting behavior, followed by those aged 26–30. In terms of education background, the mean waste-sorting behavior score of residents with college degrees was the highest, followed by scores for those with high school or technical secondary school degrees. Residents with a master’s degree or higher level of university education had the lowest mean scores in waste-sorting behavior. In terms of monthly income, residents whose monthly income ranged from 30,000 yuan to 100,000 yuan had the highest mean score for waste-sorting behavior, while residents whose monthly income exceeded 100,000 yuan had the lowest mean score, which was much lower than that for residents in other income ranges.

### 4.3. Hierarchical Regression Analysis of Individual Preference Framework and Group Preference Framework

All variables were first standardized, and then stratified regression analysis was conducted using SPSS 17.0 to separately test the regulating effects of the individual and group preference frameworks. Taking the individual preference framework as an example, job hierarchy, family ranking, household responsibilities, political status, gender, age, and monthly income as control variables. The hierarchical regression model consisted of three levels. The first level included independent variables (product facilities) and control variables (job hierarchy, family ranking, household responsibilities, political status, gender, age and monthly income). The second level included the regulatory variable (individual preference framework). The third level comprised the interaction between independent variables and regulatory variables, namely product facilities and the individual preference framework. The same hierarchical regression analysis was conducted on the group preference framework. The results of these analyses are shown in Table 4.

**Figure 2:**
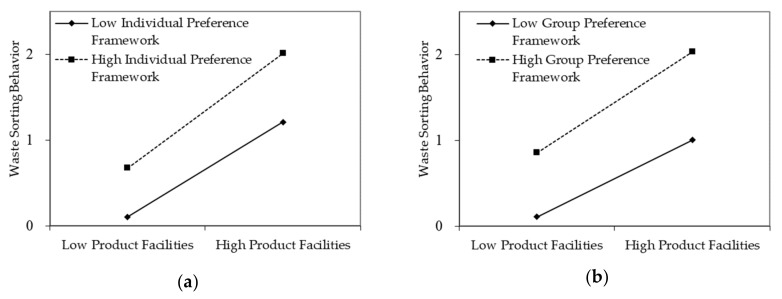
(**a**) The regulating effect of individual preference framework on waste-sorting behavior; (**b**) the regulating effect of group preference framework on waste-sorting behavior.

Both the individual preference framework and group preference framework had significant predictive effects on waste-sorting behavior, and both had a significant regulating effect on the influence path of product facilities on waste-sorting behavior. Specifically, in Model 1 (which controlled the demographic variables), the independent variable (product facility) explained 33.5% of the variance of the dependent variable (waste-sorting behavior, R^2^ = 0.335). In Model 2, the explanatory power of the model was obviously increased after the regulatory variables were introduced (R^2^ = 0.422). In Model 3, which introduced interactive terms between independent variables and regulatory variables, the explanatory power of the model increased further (R^2^ = 0.426), indicating that the individual preference framework had an obvious regulating effect on the influence path of product facilities on waste-sorting behavior. Similarly, in Model 3 of the group preference framework, the explanatory power of the model significantly increased compared with other models (R^2^ = 0.684), indicating that the group preference framework also had an obvious regulating effect on the influence path of product facilities on waste-sorting behavior. The regulating effect of the two are shown in Figure 2.

Subsequently, the regulating effects of the dimensions of the individual preference framework and group preference framework were tested. Results show that preferences for quantity, preferences for rhythm, and preferences for quality had significant regulating effects on residents’ waste-sorting behavior. When preferences for rhythm acted as the regulating variable, the R^2^ of Model 3 was higher than that when either preferences for quantity or preferences for quality were the regulating variables, indicating that the regulating effect of preference for rhythm was more significant than that of either of the other two variables. The longitudinal comparison analysis Model 3 shows that the strength of the regression of preferences for quantity (R^2^ = 0.383) was similar to that of preferences for quality (R^2^ = 0.378), indicating that the regulating effect of preferences for quantity and preferences for quality was similar. 

Regression analysis was also conducted on the various dimensions of the group preference framework. As indicated by changes in R^2^, when family preference, organizational preference, and social preference were separately used as the regulating variables, the strengths of the regressions using family preference and organizational preference were similar, indicating that the regulating effects of these two variables on waste-sorting behavior were similar. Compared with social preference, the value of R^2^ in Model 3 was greater, indicating that social preference better regulated the influence path of product facilities on waste-sorting behavior.

### 4.4. Sensitivity Analysis

In the stratified regression analysis described in Section 4.3, all the models had good significance (*p* < 0.05), and the strength of the models (R^2^), their adjusted R^2^, and changes in R^2^ were within an acceptable range. Based on the internal mechanism of regression analysis, theoretical models corresponding to the individual preference framework and its various dimensions, and corresponding to the group preference framework and its various dimensional regulatory effects were developed, as shown in Equations (1) and (2):

(1) $$Z=\beta_{1}+\lambda_{1}Y_{individual}+\mu_{1}XY_{individual}+\varepsilon_{1}$$

(2) $$Z=\beta_{2}+\lambda_{2}Y_{group}+\mu_{2}XY_{group}+\varepsilon_{2}$$

In these equations, X represents the product facilities, Y_individual_ represents the individual preference framework and its various dimensions, XY _individual_ represents the interaction between product facilities and the individual preference framework and its dimensional variables, Y_group_ represents the group preference framework and its various dimensions, XY group represents the interaction between product facilities and the group preference framework and its dimensional variables, and Z represents waste-sorting behavior. β_i_, λ_i_, and μ_i_ (i = 1, 2) represent the regression coefficients of each variable, respectively, and ε_1_ and ε_2_ are constant terms.

**Figure 3:**
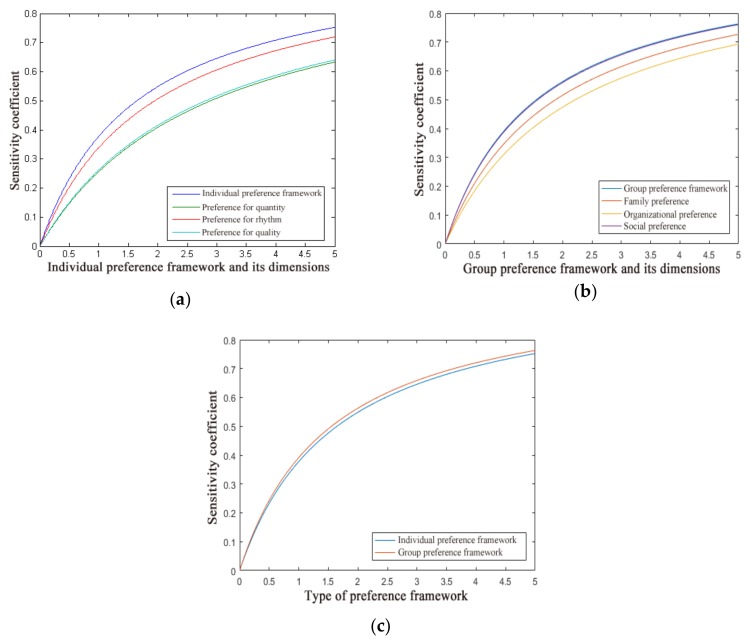
(**a**) Sensitivity of waste-sorting behavior to the individual preference framework and its dimensions; (**b**) sensitivity of waste-sorting behavior to the group preference framework and its dimensions; (**c**) sensitivity of waste-sorting behavior to the individual preference framework and the group preference framework.

To analyze and compare the regulatory effects of different variables in the path of influence of product facilities on waste-sorting behavior, a sensitivity coefficient (SAF) was used to measure the sensitivity of independent variables to dependent variables in the two theoretical models. Sensitivity analysis identifies sensitive parameters according to whether small changes in input parameters will produce large changes in dependent variables [48]. The following equation was used to calculate SAF, as shown in Equation (3):

(3) $$\mathrm{SAF}=\left(\Delta Z/Z\right)/\left(\Delta Y/Y\right)$$

where, ΔZ/Z represents the change rate of the value of the dependent variable and ΔY/Y represents the change rate of the value of the independent variable. The larger the $\left|\mathrm{SAF}\right|$, the more sensitive is the dependent variable to the independent variable; otherwise, the dependent variable is insensitive. The sensitivity coefficients of waste-sorting behavior to the individual preference framework and its dimensions, to the group preference framework and its dimensions, and to the combined networks are shown in Figure 3, respectively.

In each dimension of the individual preference framework, the sensitivity coefficient of preference for quantity was the highest, and significantly higher than the preferences for rhythms and preferences for quality. In practical terms, these results indicate that “for every unit of preference for quantity added, the impact path of product facilities on residents’ waste-sorting behavior will be more improved than for every unit of preference for rhythm or preference for quality added.” Among the dimensions of the group preference framework, the sensitivity coefficient of social preference was the highest, i.e., residents’ waste-sorting behavior was more sensitive to social preference than to other dimensions, and social preference had a stronger regulating effect on residents’ waste-sorting behavior. The overall comparison between the individual preference framework and the group preference framework showed that the sensitivity coefficient of the group preference framework was higher, so the group preference framework better regulated the influence path of product facilities on residents’ waste-sorting behavior.

## 5. Discussion

In this study, correlation analysis showed a positive correlation between product facilities and residents’ waste-sorting behavior. Many scholars also pointed out that convenient facilities and resources in the household environment have a significant direct impact on environmental behavior [49,50,51]. Additionally, the results showed that waste-sorting behavior was significantly and positively correlated with the individual preference framework and its dimensions, as well as with the group preference framework and its dimensions. This suggests that individuals’ waste-sorting behavior is influenced not only by their comfort preference [20], but also by environmental pressure, such as social norms [52], government regulation [53], and the notion of an “Exemplary Role” [25]. 

Furthermore, there was no significant gender difference in residents’ waste-sorting behavior, which was consistent with the findings of Oskamp et al. [54] and Gamba and Oskamp [55]. However, individuals of different ages, education backgrounds, and monthly incomes showed different waste-sorting behaviors. Specifically, individuals in the younger age groups exhibited better sorting behaviors (higher scores) than those in other age groups. This may be because young people in China are receiving education related to environmental protection and have better awareness about waste (including waste sorting), which is more likely to promote waste-sorting behaviors. Some scholars also attribute the better waste-sorting behavior among young people to the “Intergenerational Effect” [56]. Interestingly, respondents that had high academic qualifications did not generate high waste-sorting behavior scores. The main reason for this result may be that most university education systems do not pay enough attention to environmental protection publicity and supervision [57]. Residents with high incomes also had much lower waste-sorting behavior scores than those in other income groups. The reason for this result may be that high-income groups may focus their time and energy on creating wealth and neglect the importance of waste sorting.

Both the individual preference framework and the group preference framework had a positive regulating effect on the influence path of product facilities on waste-sorting behavior; that is, the individual and group preference frameworks could “amplify” the promoting effect of product facilities conditions on waste-sorting behavior. On the one hand, individuals who pursue a high-quality lifestyle will pay more attention to the environment around them, while those who practice a slow pace of life will be more patient than others, which will prompt them to take the initiative in waste sorting. On the other hand, when individuals are in a group environment where environmental protection is prevalent, they are more likely to have a sense of social identification for waste sorting [58], and thus, participate in waste sorting consciously.

Many scholars have found that in organizations, work preference has a regulating effect on the influence path of relevant behavioral variables. For example, Chang et al. [59] tested 1365 primary school students in Hong Kong and found that teacher preference, or the extent to which classroom teachers liked children in the classroom, regulated the relationship between social behavior and peer acceptance of children of different age groups. Gupta [60] also found that individual creative style preference significantly moderates the relationship between organizational culture and employee creative behavior. Cécile et al. [61] and Wettstein et al. [62] also found individual preference and social preference have a regulating effect on relevant behavioral variables. The findings of this study in the field of environmental behavior further verify the regulating effect of preferences on the path of environmental behavior and enrich and perfect the relevant theoretical research about preference frameworks.

Overall, the group preference framework had a better regulating effect on waste-sorting behavior than the individual preference framework. Furthermore, among the variables in each dimension of the group preference framework, the social preference variables were more able to regulate the impact path of product facilities on residents’ waste-sorting behavior than the family preference variables and the organizational preference variables. In other words, the group preference framework had a more significant regulating effect, and the social preference variable was the most “prominent” of its sub-variables. On the one hand, many people believe that only strong institutions can effectively solve environmental problems, especially in government-led countries such as China [63]. Thus, the impact of the overall social environment on Chinese residents is much higher than that of families and organizations. In other words, under the influence of a framework of social preference, individuals are more willing to follow the government’s guidance and actively participate in waste-sorting activities. Of course, this situation may also be applicable to other countries; Yokoo et al. [64] confirmed that social preference is an important determinant of informal household recycling behavior in developing countries. On the other hand, under the influence of group psychology, people are likely to exhibit a behavior that can be called “following the crowd” [65]. When most people in a society are consciously engaged in waste sorting, other people will also participate. On the contrary, if few or no people in society are involved in waste sorting, other people will not take the initiative to practice waste sorting even in the presence of good product facilities. Therefore, social preference variables play a crucial role in the influence path of product facilities on residents’ waste-sorting behavior. This finding not only enriches the theoretical research in the field of environmental management, but also provides a new direction for the policy guidance regarding waste sorting.

## 6. Conclusions

Based on preference framework theory, this study explored the relationship between product facilities and residents’ waste-sorting behavior. Through the investigation and analysis of China’s urban product facilities, preference frameworks and residents’ waste-sorting behavior, the promotion effect of individual preference framework variables and group preference framework variables on residents’ waste-sorting behavior was found. The study results support the following conclusions.

There is no significant difference in residents’ waste-sorting behavior based on gender, but there are significant differences based on the age, education background, and monthly income of residents. Moreover, the group preference framework and its various dimensions have significant regulatory effects on residents’ waste-sorting behavior. A group preference framework is more able to regulate the impact path of product facilities on residents’ waste-sorting behavior than an individual preference framework. Particularly, among the sub-variables in a group preference framework, the social preference variable is the most “prominent” regulatory variable. 

The results of this study provide important theoretical support and a practical basis for promoting urban residents’ participation in waste-sorting activities. This knowledge should help cities to improve waste management as soon as possible. These findings have policy implications, and the following actions are proposed to address these implications.

(1) Consolidate the sorting foundation, optimize the existing product facilities, and create a good waste sorting environment.

Good product facilities are conducive to residents’ active participation in waste-sorting activities. Therefore, in China, environmental protection-related infrastructure construction needs to be solved urgently. First, the principle of convenience should be considered when developing waste-sorting facilities. As far as possible, waste-sorting facilities should not be too complicated. The layout and cleaning frequency of waste receptacles should be adjusted to local conditions in consideration of residents’ needs for space and the value of their time. Furthermore, the upgrading of waste-sorting facilities should be completed as soon as possible. At present, there is still a large number of waste-sorting equipment using “asynchronous” sorting processes in streets, residential areas, and residential buildings, and there are no sorting facilities that are matched with the waste-sorting system. As a result, “temporary stacking areas” easily develop, which make it difficult for residents to further sort waste when discarding it. If not addressed in a timely manner, these areas cause secondary pollution. Therefore, it is urgent that facilities to support waste sorting be implemented. In addition, important measures to reduce the generation of municipal solid waste at the source should be implemented, such as promoting and popularizing green products, promoting technological innovations in environmentally friendly packaging, developing degradable packaging materials and new environmentally friendly lunch boxes, and focusing on accelerating the research and development of “green” core products and other key waste management technologies.

(2) Strengthen the sorting preference of multiple subjects, build an integrated and comprehensive sorting mechanism of “Government Taking the Lead, Organization Demonstration, Street Promotion and Family Influence”, and make the sorting concept widely popular.

Whether residents actively participate in waste-sorting activities is not only influenced by their individual preferences, but also closely related to the group environment. To strengthen the sorting preference of private residents, government personnel, those working in institutions and other work units, as well as those in communities, streets, and families, a systematic waste-sorting system should be established. First, the government should take the lead, vigorously promoting the desirable atmosphere of waste-sorting, and implementing measures related to waste sorting. The government should establish an environmental supervision mechanism led by urban management departments with the participation of multiple departments, and impose certain punishment measures on those who do not implement waste sorting. Second, public institutions should try their best to demonstrate waste-sorting practices, forming a good sorting atmosphere. Particularly, organizations such as government agencies, public institutions, and schools should make “waste sorting” one of their performance criteria, urge employees and students to take the initiative in waste sorting, and strengthen individual awareness of sorting and sense of environmental responsibility. Third, opportunities should be pursued to expand the waste sorting and promotion effect of major public places and street communities. In view of the large number of shopping malls, transportation stations, libraries, parks, scenic locations, and other places with a large amount of traffic, opportunities exist to optimize the waste sorting and recycling service process, establish a sound waste sorting and rewards and punishment system, implement publicity and supervision of mobile groups, and clarify the sorting responsibilities of individuals. Fourth, the impact of household waste sorting should be enhanced by providing effective waste-sorting and recycling containers, and guiding family members to sort waste at the source. 

## Figures and Tables

**Table 1:** Sample scales.

Content	Item Description	Not Conformed → Quite Conformed				
Product facilities	In daily work and life, the waste can I see can guide me to sort the waste.	1	2	3	4	5
Individual preference framework	I care so much about quality of life that I never compromise on it.	1	2	3	4	5
Group preference framework	My family think we should sort the waste.	1	2	3	4	5
Waste-sorting behavior	It is my habit to sort waste.	1	2	3	4	5

**Table 2:** Sample distribution of formal survey.

Demographic Variable		Frequency (*N*)	Proportion (%)	Demographic Variable		Frequency (*N*)	Proportion (%)
Age (years old)	≤17	59	3.9%	Gender	male	789	52.4%
	18–25	424	28.2%				
	26–30	406	27.0%		female	716	47.6%
	31–40	324	21.5%				
	41–50	210	14.0%	Monthly income (dollar)	≤288	271	18%
	≥51	82	5.4%		289–576	272	18.1%
Education background	Junior high school or below	100	6.6%		577–864	337	22.4%
	High school or technical secondary school	203	13.5%		865–1152	227	15.1%
	Junior college	238	15.8%		1153–1440	160	10.6%
	Bachelor	720	47.8%		1441–4322	150	10.0%
	Master or above	244	16.2%		> 4322	63	4.2%

**Table 3:** Analysis of the influence of gender, age, education background, and monthly income on waste-sorting behavior of urban residents.

Variable		Mean	F	Significance	Variable		Mean	F	Significance
gender	male	3.248	0.128	0.721	age (years old)	≤17	3.524	1.988	0.045
	female	3.179				18–25	3.145		
Monthly income (dollar)			5.573	0.000		26–30	3.209		
	≤288	3.137				31–40	3.232		
	289–576	3.296				41–50	3.272		
	577–864	3.218				≥51	3.343		
	865–1152	3.207			education background	Junior high school and below	3.211	10.909	0.000
	1153–1440	3.108				High school or technical secondary school	3.458		
	1441–4322	3.315				Junior college	3.470		
	> 4322	3.692				Bachelor	3.199		
	≤288	2.257				Master or above	3.090		

**Table 4:** Regulating effects of the individual preference framework and the group preference framework on the influence path of product facilities on waste-sorting behavior.

Model	Model 1			Model 2			Model 3		
variable	B	Standard error	T value	B	Standard error	T value	B	Standard error	T value
Constant term	0.209	0.148	1.410	0.068	0.138	0.490 ***	0.048	0.138	0.347
PF	0.876	0.033	26.423 ***	0.609	0.036	17.072 ***	0.610	0.036	17.159 ***
IPF				0.345	0.023	15.075 ***	0.342	0.023	14.957 ***
PF × IPF							0.058	0.019	3.028 ***
R^2^	0.335			0.422			0.426		
F	94.011 ***			121.456 ***			110.824 ***		
Constant term	0.209	0.148	1.410	0.317	0.133	2.380 *	0.280	0.133	2.107 *
PF	0.876	0.033	26.423 ***	0.518	0.035	14.667 ***	0.518	0.035	14.709 ***
GPF				0.431	0.023	18.890 ***	0.444	0.023	19.302 ***
PF × GPF							0.070	0.019	3.636 ***
R^2^	0.578			0.680			0.684		
F	94.011***			143.092 ***			131.158 ***		

Note: * means *p* < 0.5, *** means *p* < 0.001. PF indicates product facilities, IPF indicates individual preference framework, GPF indicates group preference framework.

## Appendix A

**Table A1:** The scale items for product facilities and preference framework and waste-sorting behavior.

Dimensions	Items Descriptions
Product Facilities	I think almost all the daily products that can be consumed at this stage are non-recyclable.
	I think many product packaging does not clearly indicate what kind of waste it belongs to.
	I think our country’s waste-sorting technology is lacking at this stage.
	In daily work and life, the waste bin beside me is placed in a reasonable position.
	In daily work and life, the signs on the waste can express clear meaning.
	In my daily work and life, the waste cans I see can guide me to sort the waste.
Preferences for Quantity	I believe in a word called “the more the better.”
	I like the celebration of big scenes, with many people.
	I like the life with high consumption level.
Preferences for Rhythm	I like fast-paced life.
	I like to finish the task as soon as possible.
	I like to do many things at the same time.
	I will make good plans every day to avoid inefficiency and wasting time.
Preferences for Quality	I value the internal quality of products more than other factors.
	I am very particular about the quality of life and I never make do with it.
	As long as I can, I will make myself comfortable.
Family Preference	My family think waste should be sorted.
	My family think it is commendable to sort the waste.
	My family think littering is a disgrace.
Organizational Preference	My colleague think that waste should be sorted.
	My colleagues think it is commendable to sort the waste.
	My colleague think littering is a disgrace.
Social Preference	People in my area think that waste should be sorted.
	People in my area think it is commendable to sort the waste.
	People in my area think littering is a disgrace.
Waste-Sorting Behavior	It is my habit to sort waste.
	I just like littering.
	I didn’t know there was waste sorting.
	I think waste sorting can improve the living environment of myself and my family, so I will sort it out.
	I think waste sorting can earn economic benefits, so I will sort it out.
	I think waste sorting is good for my health, so I will sort it out.
	Since everyone sorts waste, I also sort the waste.
	Since waste sorting can enhance my image, I will sort it out.
	I will not litter because I am afraid of being looked at differently.
	I always advise people around me to sort the waste.
	For people or units with improper waste disposal, I will promptly report to the relevant departments
	I take an active part in civic meetings related to waste sorting.
	I take an active part in various group activities that can promote garbage classification.
	I actively participate in the formulation of garbage classification policies and standards
